# GP73 regulates Hepatic Steatosis by enhancing SCAP-SREBPs interaction

**DOI:** 10.1038/s41598-017-06500-9

**Published:** 2017-11-02

**Authors:** Xiaoli Yang, Feixiang Wu, Jiankang Chen, Cui Wang, Yongjie Zhu, Feng Li, Qinfang Hao, Cuijuan Duan, Li Wang, Xueping Ma, Deyong Zou, Li Luo, Yiwen Zhao, Kai Guan, Yuan Cao, Pingping Zhang, Pengyu Zhou, Shengli Ma, Zhifeng Yan, Jia Li, Yanhong Zhang, Congwen Wei, Hui Zhong

**Affiliations:** 1 The General Hospital of Chinese People’s Armed Police Forces, Beijing, 100039 China; 2 Institute of Health Sciences, Anhui University, Hefei, 230601 China; 3 State key Laboratory of Pathogen and Biosecurity, Beijing Institute of Biotechnology, Beijing, 100850 China; 4 Department of Hepatobiliary Surgery, Affiliated Tumor Hospital of Guangxi Medical University, Nanning, 530021 China; 5 Department of Laboratory Medicine, The General Hospital of Jinan Military Region, Jinan, Shandong 250031 China; 6 The General Hospital of Chinese People’s Liberation Army, Beijing, 100039 China; 7 Shaanxi Provincial People’s Hospital, Xi’an, 710068 China

## Abstract

Elevated Golgi phosphoprotein 2 (GP73, also known as GOLPH2 or GOLM1) expression in serum and liver, which can be induced by viral infection and cytokine treatments, is intimately connected with liver disease, including acute hepatitis, cirrhosis and hepatocellular carcinoma (HCC). However, its pathogenic roles in hepatic diseases have never been clarified in detail. Here, we showed that the upregulated GP73 is indispensable for SREBPs activation and lipogenesis. Notably, GP73 overexpression enhanced SCAP-SREBPs binding and its Golgi trafficking even under cholesterol sufficiency. Consistent with these functional findings, GP73 blockage could alleviate tunicamycin-induced liver steatosis by reducing SREBPs activation. A significant positive correlation of GP73 with genes in lipid metabolism pathway was also identified in liver cancer based on data from The Cancer Genome Atlas (TCGA) dataset. Our findings revealed previously unrecognized role of GP73 in lipid metabolism.

## Introduction

GP73, which is also named Golgi membrane protein (GOLM1) or Golgi phosphoprotein 2 (GOLPH2), is a type II transmembrane protein located on *cis* and medial-Golgi. GP73 consists of a short N-terminal cytoplasmic domain, a transmembrane domain and a larger C-terminal domain located in the luminal surface of the Golgi apparatus. The C-terminal domain is predicted to contain two helical regions, each 150 to 200 residues in length^1^. Mutation study establishes that the N-terminal region and its transmembrane domain determine its Golgi localization^2^. The C-terminal domain contains putative protein interaction motif. Its truncation reduces mice cumulative survival rate with varying degrees of renal disease, and marked microvascular hepatic steatosis^3^. Immunohistochemical studies demonstrate that GP73 is preferentially expressed in normal epithelial cells of various human tissues, but not in hepatocytes^4^. However, in diseased livers or upon virus infection, hepatocyte expression of GP73 is dramatically upregulated^4,5^. Elevated GP73 expression is not only observed in acute hepatitis and during the progression of liver disease to cirrhosis^6^, but also in many cancers including lung cancer^7^, hepatocellular carcinoma (HCC)^2^, gastric cancer^8^, and prostate cancer^9^. Cytokines are confirmed to play an important role in GP73 expression regulation, with interferon gamma (IFN-γ) upregulating, while tumor necrosis factor alpha (TNF-α) downregulates^5^. Both intracellular and secreted GP73 exist as coiled-coil dimers^2^. Using yeast two-hybrid screening, secretory clusterin (sCLU) is shown to bind the coiled-coil domain of GP73^10^. Details about the regulation of GP73 expression and its exact functions remain elusive.

SREBPs are believed to be the master transcriptional regulators of lipid homeostasis^11^. Mammalian cells produce three SREBP isoforms, SREBP-1a, SREBP-1c, and SREBP-2. SREBP-1c predominantly activates genes involved in the synthesis of fatty acids and their incorporation into triglycerides and phospholipids. SREBP-2 is relatively specific to genes required for the uptake and biosynthesis of cholesterol. The SREBP-1a seems to be involved in both pathways^11^. The inactive precursors of SREBPs reside in ER membranes bound with SREBP cleavage-activating protein (SCAP). Sterols induce binding of SCAP to Insig-1, and prevent SREBP/SCAP from exiting the ER^12^. Upon cellular sterol depletion, the SREBP/SCAP complex is translocated to the Golgi *via* COP-II-coated vesicles. There, SREBPs undergo two proteolytic processes, first by S1P, then S2P. As a result, the active SREBPs transcription factors are released and enter nucleus to regulate the transcription of the target genes^13^. In the cholesterol and triglyceride biosynthesis pathways, the active SREBP transcription factors trigger the expression of genes that encode cholesterogenic or lipogenic enzymes, thus promoting lipid synthesis. Upon inhibition of ER stress and SREBPs’ proteolytic cleavage, both hepatic steatosis and insulin sensitivity are improved^14^. Sterol regulates the association between the SREBP/SCAP complex and Insig by binding directly to Insig or to the sterol sensing domain of SCAP.

In this study, for the first time, we have provided evidence that GP73 functions to mediate SREBPs proteolytic cleavage. The involvement of GP73 in promoting SCAP stabilization, SCAP Golgi localization and the increased formation of SCAP-SREBP complex regardless of cholesterol content implicates its pathological roles in hepatic steatosis and HCC progression.

## Results

### GP73 regulates the transcriptional activity of SREBP1 and lipogenesis

**Figure 1:**
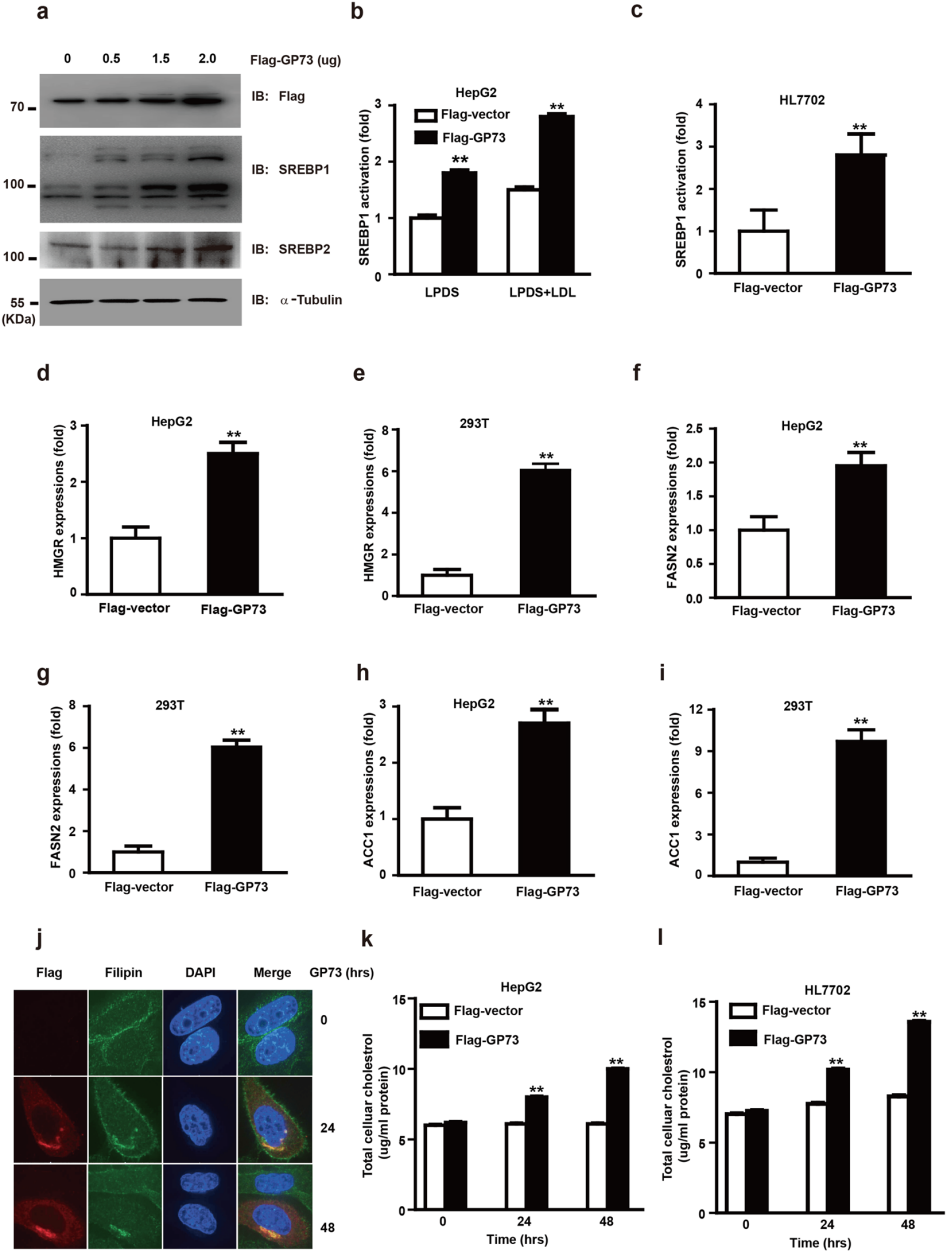
GP73 regulates the transcriptional activity of SREBPs and lipogenesis. (**a**) Immunoblotting analysis of SREBPs activation in HepG2 cells transfected with Flag-GP73 at the indicated doses. α-Tubulin was used as equal loading control. (**b**,**c**) SREBP-1 promoter activity in HepG2 (**b**) or HL7702 (**c**) cells transfected with Flag-vector or Flag-GP73 under conditions of sterol depletion or repletion. The luciferase activity was measured 36 hrs post transfection. The value was normalized with the corresponding transfection efficiency. (**d**,**f**,**h**) QRT-PCR analysis of HMGR (**d**), FASN2 (**f**), and ACC1 (**h**) mRNA abundance in HepG2 cells transfected with Flag-vector or Flag-GP73 for 24 hrs. (**e**,**g**,**i**) QRT-PCR analysis of HMGR (**e**), FASN2 (**g**), and ACC1 (**i**) mRNA abundance in 293T cells transfected with Flag-vector or Flag-GP73 for 24 hrs. (**j**) Fluorescence microscopy of Filipin staining in HepG2 cells transfected with Flag-GP73. Cells were collected at indicated hrs post transfection. (**k**,**l**) Amplex Red cholesterol assay of cellular cholesterol concentrations in HepG2 (**k**) or HL7702 (**l**) cells transfected with Flag-vector or Flag-GP73. Cells were collected at indicated hrs post transfection. Values were normalized to total cell proteins from control cells transfected with Flag-vector. Cell-based studies were performed at least three independent times with comparable results. Data represent mean ± SEM. Student’s t test was used for statistical analysis: **p < 0.01.

To decipher the possible outcome of GP73 upregulation, we used microarray analysis to determine the expression of genes altered by GP73 overexpression. Our unpublished data indicated that cholesterol pathway were ranked as the one of top 8 upregulated signaling pathways in GP73 overexpressed HepG2 cells, which propelled us to study the effect of GP73 expression on SREBPs activation. As was shown in Fig. 1a, overexpression of GP73 strongly induced SREBP-2 and SREBP-1a activation. Reporter assays showed that GP73 introduction dramatically potentiated the activation of SREBP-1a promoter in HepG2 cells and HL7702 cells regardless of cholesterol contents (Fig. 1b,c and Supplementary Fig. S1). To further elucidate SREBPs activation in HepG2 cells and 293T cells, we used QRT-PCR to determine the effect of GP73 overexpression on the mRNA levels of HMGR, HMGSC1, HMGSC2, FASN2, ACSS2 and ACC1, five genes involved in cholesterol biosynthesis motivated by SREBP-2, SREBP-1a or both. Compared with the control, the mRNA levels for HMGR (Fig. 1d,e), HMGSC1 (Supplementary Figs S3 and S4), HMGSC2 (Supplementary Figs S5 and S6), FASN2 (Fig. 1f,g), ACSS2 (Supplementary Figs S7 and S8) and ACC1 (Fig. 1h,i) were significantly increased in GP73-overexpressed HepG2 and 293T cells.

Consistent with the transcriptional activation of SREBPs target genes, GP73 overexpression caused a substantial increase in the accumulation of intracellular cholesterol revealed by Filipin staining (Fig. 1j and Supplementary Fig. S2). At 48 hrs post GP73 transfection, cellular cholesterol levels in HepG2 cells and HL7702 cells were increased by 80% compared with the controls (Fig. 1k,l). Therefore, GP73 activates the transcriptional activity of SREBP1 and promotes lipogenesis.

### GP73 Activates SREBPs via Upregulation of SCAP

**Figure 2:**
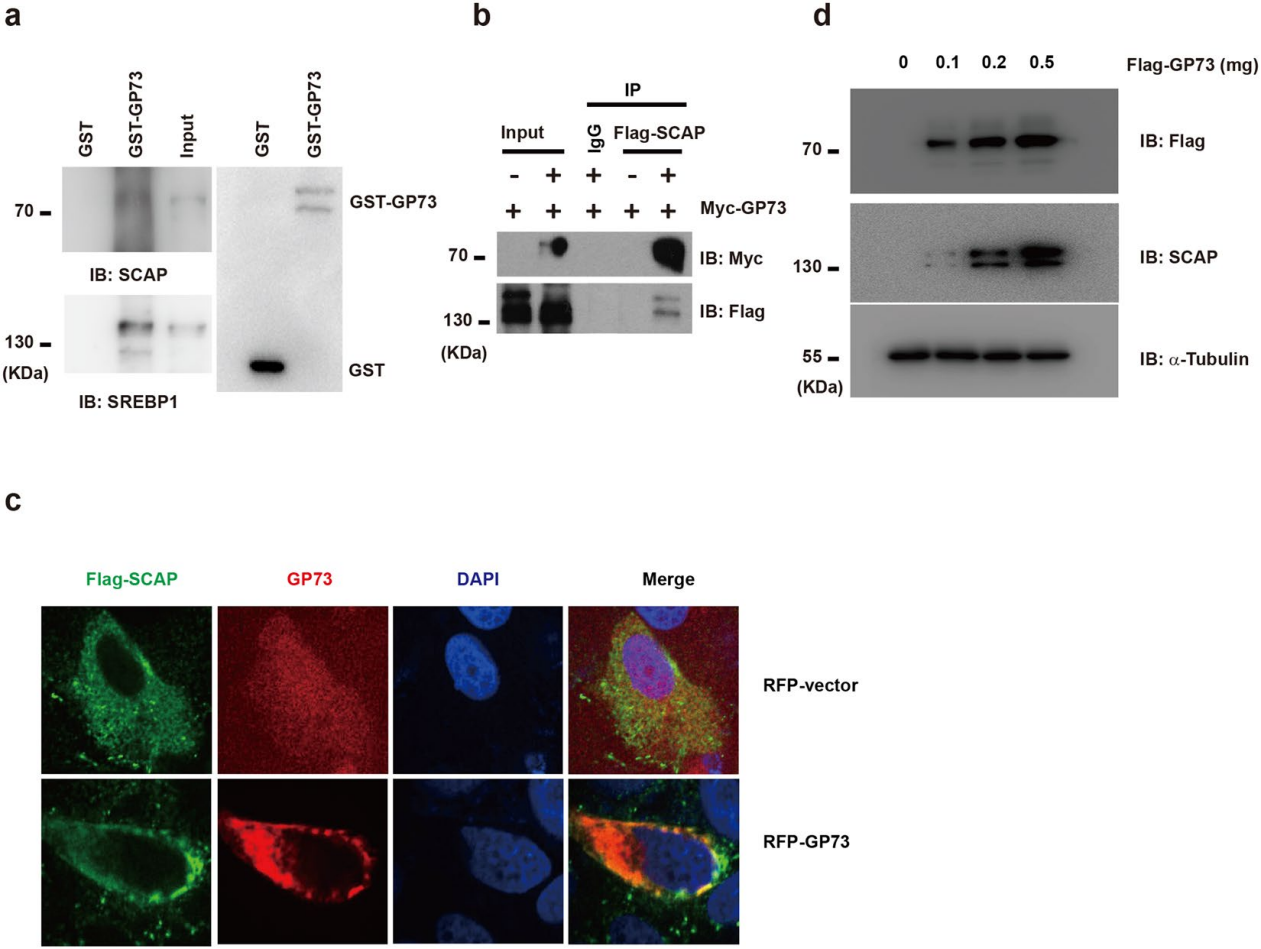
GP73 activates SREBPs *via* upregulating SCAP. (**a**) GST pull-down analysis in 293T cells transfected with GST-vector or GST-GP73. SCAP or SREBP1 antibody was used for immunoblotting assay. Loading of the GST proteins was assessed by Coomassie blue staining (bottom panel). (**b**) Immunoprecipitation analysis in 293T cells transfected with Flag-vector or Flag-SCAP in the presence of Myc-GP73. (**c**) Representative confocal immunofluorescence images of GP73 colocalized with SCAP in HeLa cells transfected with RFP-vector or RFP-GP73 in the presence of Flag-SCAP. GP73, red; SCAP, green. DAPI: blue Scale bar: 10 μm. (**d**) Immunoblotting analysis of SCAP expression in 293T cells transfected with increasing doses of Flag-GP73. α-Tubulin was used as equal loading control. Cell-based studies were performed at least three independent times with comparable results. Numbers below certain Western blots indicate relative levels determined by software-based quantification of the representative experiment shown. Data represent mean ± SEM. Student’s t test was used for statistical analysis: **p < 0.01.

SREBPs reside as transcriptionally inactive precursor proteins in the ER membrane bound with SCAP and Insigs. The SREBP/SCAP complex is translocated to the Golgi, where SREBPs are proteolytically processed to release the active SREBP transcription factors. The Golgi localization of GP73 raised the possibility that GP73 might physically interact with SCAP and or SREBPs. To test this hypothesis, we analyzed the association of GP73 with SCAP. Lysates from 293T cells were incubated with GST or GST-GP73 fusion protein. We found that both SCAP and SREBP1 bind to GST-GP73 but not to GST (Fig. 2a), demonstrating an *in vitro* interaction of GP73 with SCAP and SREBP1. Flag-SCAP was transfected together with Myc-GP73. Immunoblotting analysis of anti-Flag immunoprecipitates with anti-Myc showed a significant association between Myc-GP73 with Flag-SCAP (Fig. 2b). Immunofluorescence results also showed that transient transfected-GP73 co-localized with SCAP even under conditions of cholesterol sufficiency (Fig. 2c).

To explore further the mechanism behind the stimulatory effect of GP73 on lipid metabolism, we examined the effect of GP73 on endogenous SCAP abundance. When expression plasmids encoding GP73 were transfected into HepG2 cells, a remarkable increase in the abundance of endogenous SCAP was detected along with overexpressed GP73. This upregulation of SCAP by GP73 was dose dependent (Fig. 2d).

**Figure 3:**
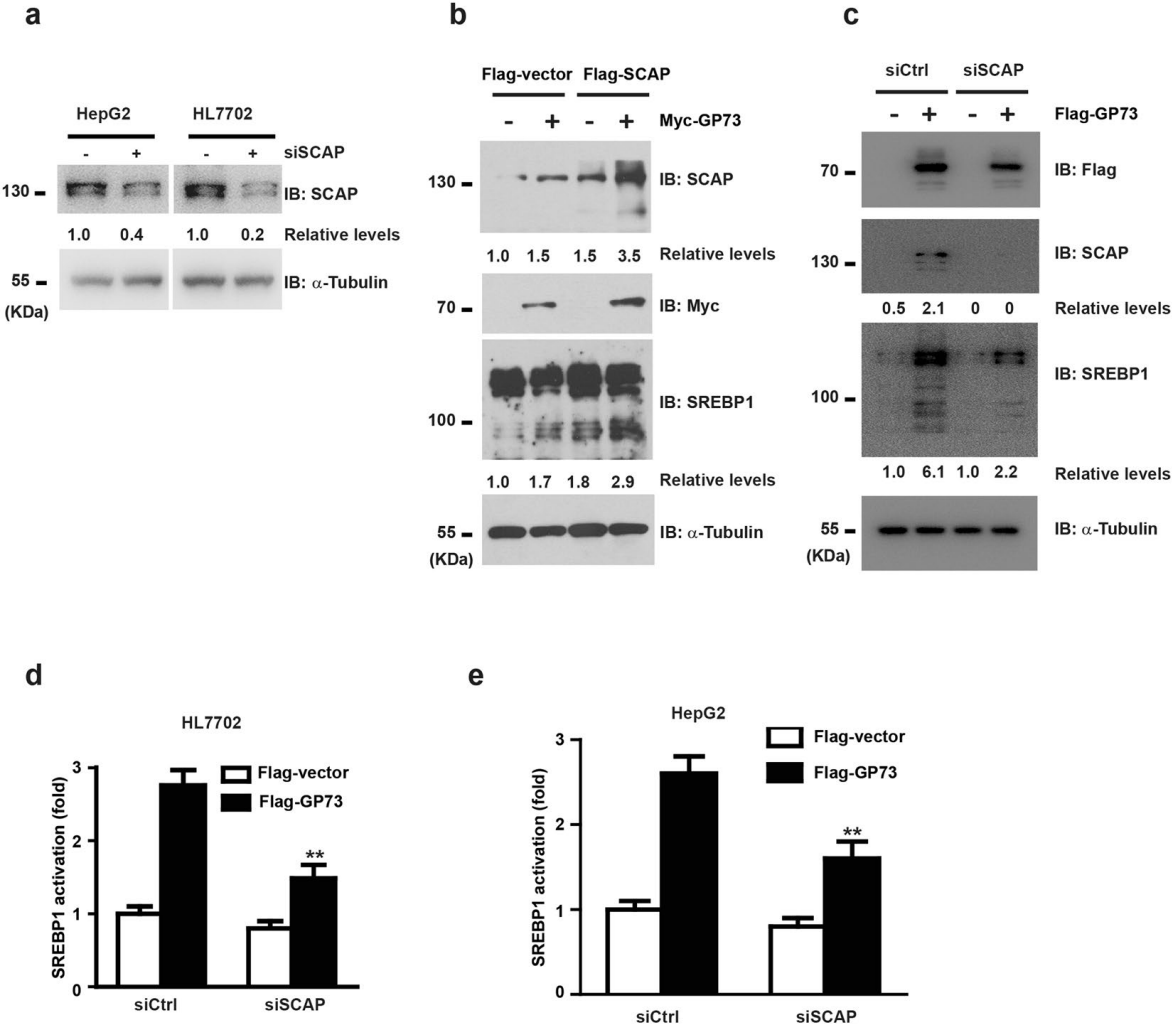
GP73 Activates SREBPs via Upregulation of SCAP. (**a**) Immunoblotting analysis of SCAP protein in HepG2 or HL7702 cells transfected with scrambled siRNA or SCAP-specific siRNA duplexes. α-Tubulin was used as equal loading control. (**b**) Immunoblotting analysis of SREBP1 activation in Flag-vector or Flag-SCAP HepG2 cells transfected with Myc-vector or Myc-GP73. α-Tubulin was used as equal loading control. (**c**) Immunoblotting analysis of SREBP1 activation in siSCAP knockdown or control HepG2 cells transfected with Flag-vector or Flag-GP73. α-Tubulin was used as equal loading control. (**d**,**e**) SREBP-1 promoter activity in siSCAP knockdown or control HL7702 cells and HepG2 cells transfected with Flag-vector or Flag-GP73. The luciferase activity was measured 36 hrs post transfection. Data was normalized based on transfection efficiency. Cell-based studies were performed at least three independent times with comparable results. Numbers below certain Western blots indicate relative levels determined by software-based quantification of the representative experiment shown. Data represent mean ± SEM. Student’s t test was used for statistical analysis: **p < 0.01.

We then tried to delineate the effect of SCAP upregulation by GP73 on SREBP1 activation. Firstly, we tested the efficiency of SCAP knockdown in HepG2 and HL7702 cells (Fig. 3a). Notably, SCAP introduction led to significantly higher SREBP1 cleavage in cells with overexpressed GP73 (Fig. 3b). Furthermore, the activation of SREBP1 by GP73 overexpression under cholesterol sufficiency could be abolished by SCAP siRNA knockdown (Fig. 3c). Consistent with this finding, GP73 lost its ability to activate SREBP1-luc promoter in SCAP siRNA knockdown HepG2 and HL7702 cells (Fig. 3d,e).

### GP73 promotes SCAP Trafficking to the Golgi and increases SCAP-SREBP association

**Figure 4:**
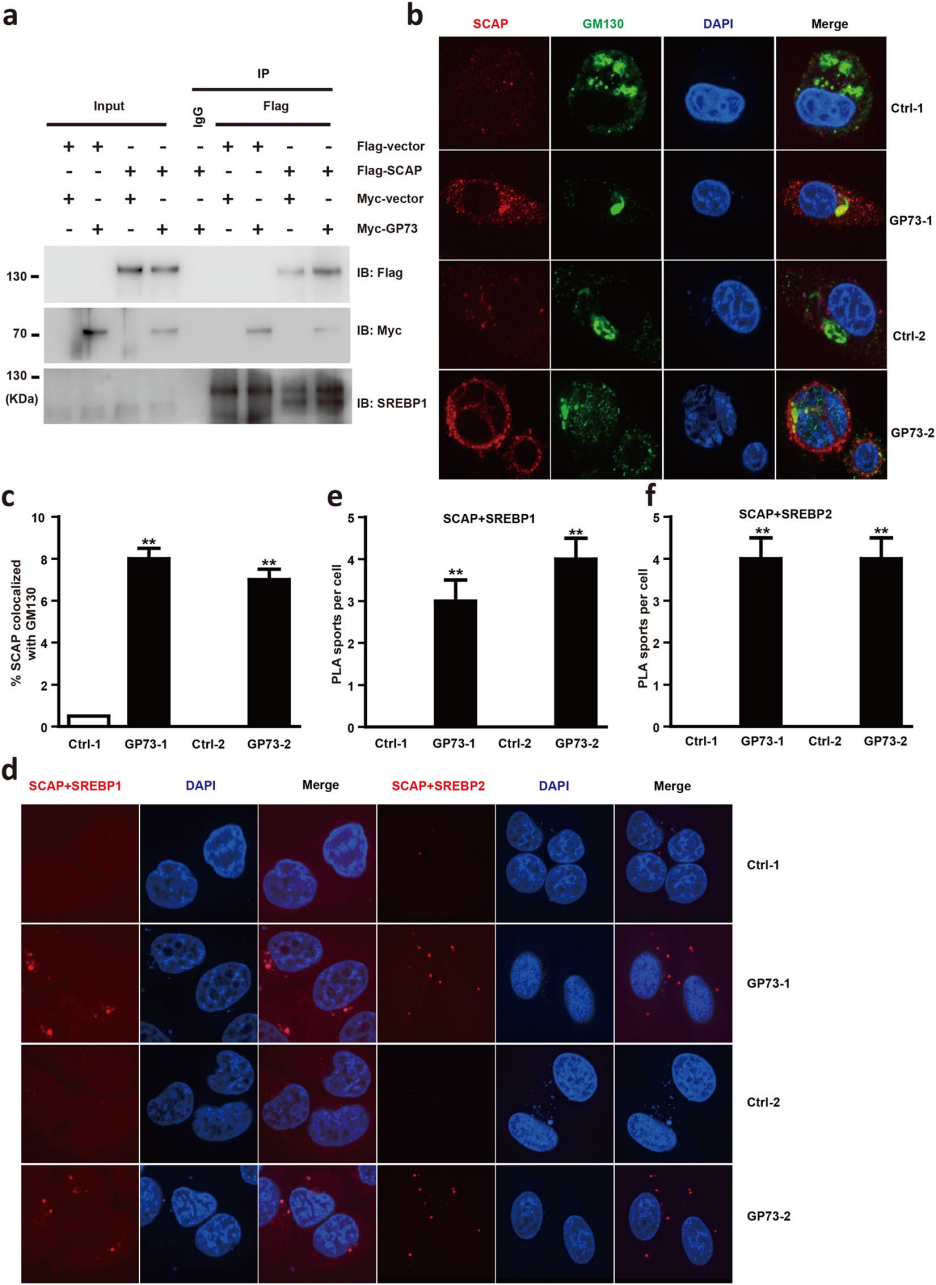
GP73 promotes SCAP Trafficking to the Golgi and increases SCAP-SREBP association. (**a**) Immunoprecipitation analysis of SCAP-SREBP1 association in Flag-vector or Flag-SCAP HepG2 cells transfected with Myc-vector or Myc-GP73. (**b**) Representative confocal immunofluorescence images of SCAP localized in Golgi apparatus in Ctrl-1, Ctrl-2, GP73-1, and GP73-2 cells. SCAP, red; GM130, green. DAPI: blue Scale bar: 10 μm. (**c**) Quantification of confocal immunofluorescence images signals shown in B panel. (**d**) *In situ* PLA assay of SCAP-SREBP1 and SCAP-SREBP2 complex in Ctrl-1, Ctrl-2, GP73-1, and GP73-2 cells. SCAP-SREBP1 or SCAP-SREBP2 complex, red; nuclei, blue. (**e**,**f**) Quantification of PLA signals shown in D panel. Cell-based studies were performed at least three independent times with comparable results. Data represent mean ± SEM. Student’s t test was used for statistical analysis: **p < 0.01.

Our studies have shown that GP73 stimulates SREBP1 activation by upregulating SCAP, indicating that GP73 might affect the kinetics of the SCAP-SREBP1 association even under conditions of cholesterol sufficiency. 293T cells were cotransfected with Flag-SCAP along with the expression vector encoding Myc-GP73 or an empty vector. Our results indicated that SCAP-SREBP1 interaction was greatly enhanced with the addition of GP73 (Fig. 4a). Two stable cell lines (GP73-1 and GP73-2) were constructed, which showed increased GP73 expression in Golgi compartment (Supplementary Figs S9 and S11). Due to the importance of SCAP Golgi localization on the activation of SREBPs, we next assessed the involvement of GP73 in the Golgi localization of SCAP. As expected, the staining of SCAP was greatly enhanced in GP73 overexpressing cells (Supplementary Fig. S10). Interestingly, SCAP displayed primarily cytosolic distribution in the control cells, whereas GP73 introduction led to SCAP redistribution in Golgi compartments (Fig. 4b,c).

In addition, we wanted to visualize SCAP-SREBP1 complex formation by an *in situ* proximity ligation assay (PLA) upon GP73 overexpression. We observed a few spots representing SCAP-SREBP1 complex in 293T cells, and the number of spots were greatly increased in GP73 overexpressed cells (Fig. 4d,e). We also found an increased number of SCAP-SREBP2 complexes in GP73 overexpressed cells (Fig. 4d,f). These results suggest that GP73 promotes the formation of the SCAP-SREBPs complex to stimulate lipogenesis even in cholesterol sufficiency, implicating the pathogenic roles it might play in hepatic steatosis.

### GP73 regulates steatosis under pharmacologic ER Stress *in vivo*

**Figure 5:**
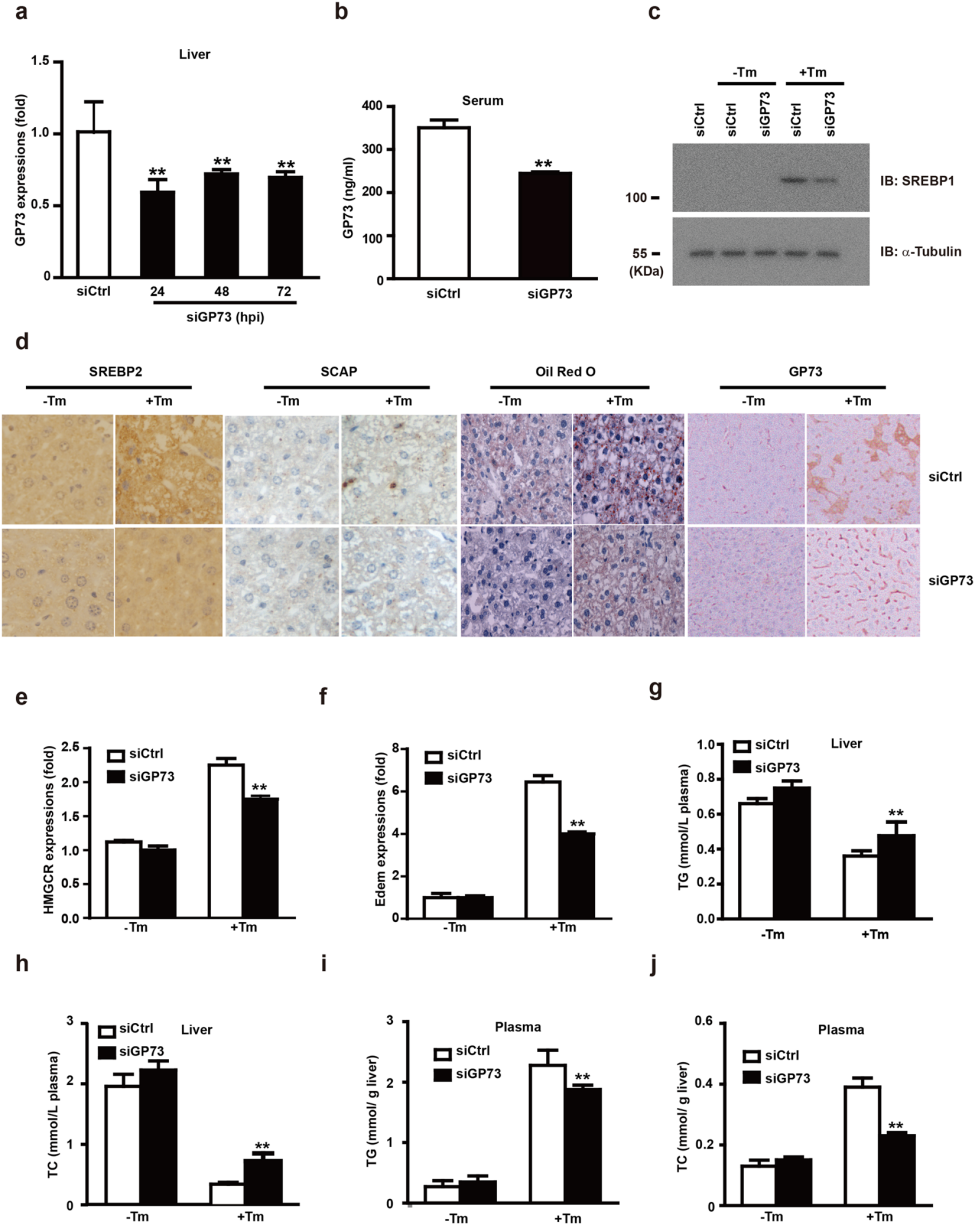
GP73 regulates steatosis under pharmacologic ER Stress *in vivo*. (**a**) QRT-PCR analysis of GP73 mRNA expression in the liver tissues of mice injected with scrambled siRNA or GP73-specific siRNA duplexes at three time points. (**b**) The level of GP73 expression in the serum of mice injected with scrambled siRNA or GP73-specific siRNA duplexes for the indicated time intervals. (**c**) Immunoblotting analysis of SREBP1 activation in mice liver tissues after injection with scrambled siRNA or GP73-specific siRNA duplexes and challenged with Tm or vehicle. (**d**) SREBP2, SCAP and Oil Red O staining of mice liver tissues after injection with scrambled siRNA or GP73-specific siRNA duplexes and challenged with Tm or vehicle. (**e**,**f**) QRT-PCR analysis of HMGR (**e**), Edem (**f**) mRNA abundance in the liver tissues of mice injected with scrambled siRNA or GP73-specific siRNA duplexes three times at 8 h intervals and challenged with Tm or vehicle control for 8 hrs at 24 hpi (hrs post injection). (**g**,**h**) Levels of TG (**g**) and TC (**h**) in mice plasma after injection with scrambled siRNA or GP73-specific siRNA duplexes and challenged with Tm or vehicle. (**i**,**j**) Levels of TG and TC in mice liver after injection with scrambled siRNA or GP73-specific siRNA duplexes and challenged with Tm or vehicle. Cell-based studies were performed at least three independent times with comparable results. Data represent mean ± SEM. Student’s t test was used for statistical analysis: **p < 0.01.

To further explore pathophysiologic roles of GP73 in ER stress-induced steatosis, GP73 RNAi duplexes (siGP73), which were designed to knockdown GP73, were introduced into mice hepatocytes via hydrodynamic tail vein injection. The mRNA levels for GP73 at 24, 48 and 96 hrs after injection were analyzed. As shown in Fig. 5a, the mRNA levels of GP73 were decreased by about 40% 24 hrs after injection and remained at similar level until the last time point tested. Serum GP73 protein levels were also reduced at 24 hrs after injection (Fig. 5b). Therefore, we chose 24 hrs post injection to perform NASH induction in mice to study the effect of GP73 deficiency on SREBPs activation in Tm-treated mouse liver^15^. The level of SREBP-1c cleavage (Fig. 5c), the staining intensity of SREBP2 and SCAP (Fig. 5d) in the liver of siGP73 mice were all significantly reduced compared to that of the wild-type mice at day 1 post infection. As a consequence of this drop in hepatic SREBP-1c activation in siGP73 mice, the mRNA levels of HMGCR and Edem were induced at much lower amounts in siGP73 mice comparing with the control mice (Fig. 5e,f).

Next, we determined whether GP73 can modulate steatosis induced by acute ER stress in mice. As was shown in Fig. 5g,h, Tm-induced hepatic TG and TC accumulation were also greatly reduced in siGP73 mice. On the other hand, the detected amount of circulating plasma triglycerides (Fig. 5i) and cholesterol (Fig. 5j) in the blood was increased. Liver staining revealed large lipid droplets in Tm-challenged mice, while GP73 knockdown liver had lipid droplets considerably decreased in size and number (Fig. 5d). Therefore, blockage of GP73 expression could alleviate liver steatosis by reducing SREBPs activation.

### GP73 is positively correlated with metabolism pathway in patients with liver cancer

**Figure 6:**
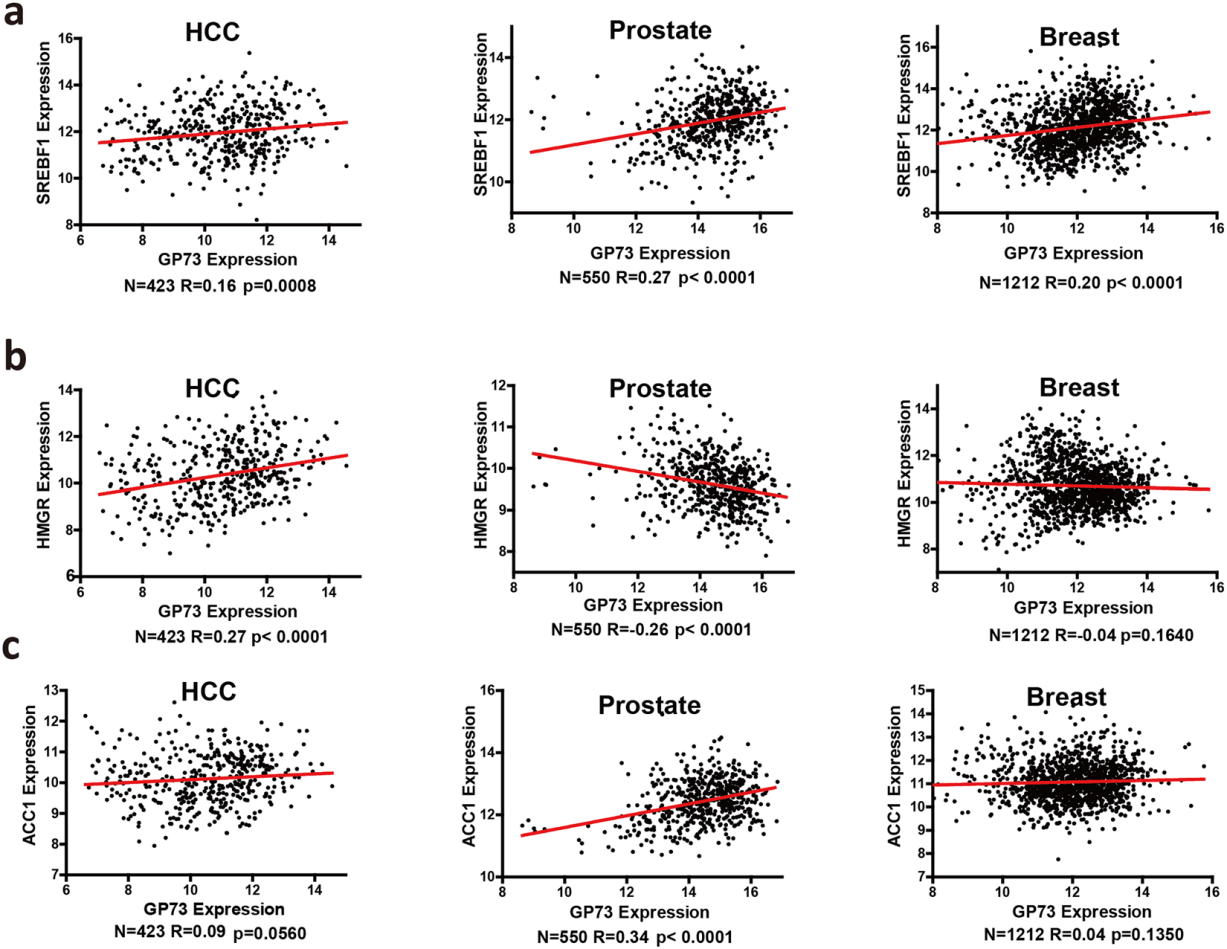
GP73 is positively correlated with metabolism pathway in patients with liver cancer. The correlation between GP73 expression with SREBP1 (**a**), HMGR (**b**) and ACC1 (**c**) in human liver (n = 423), prostate (n = 550) and breast (n = 1212) cancer samples from the TCGA data set.

Since GP73 upregulation connects with the metabolism pathway, and deregulation of this pathway will result in cancer, we thus analyzed the association of GP73 with genes involved in this pathway. The data were selected from The Cancer Genome Atlas (TCGA) for a range of cancers. We then evaluated the association between GP73 expression and the expression of genes associated with metabolism pathway (SREBF1, HMGR, and ACC1). Significant correlation between GP73 expression and each of the 3 genes in HCC was identified (Fig. 6a–c). 2 out of 3 in genes in prostate cancer and 1 out of 3 analyzed genes in breast cancer showed positive correlation with GP73 expression (Fig. 6a–c).

## Discussion

GP73 is expressed in both normal and diseased human tissues of the epithelial lineage and is essential for normal liver and kidney functions. GP73 may assist in protein transportation and secretion. However, the detailed mechanism for GP73 function is unknown. In this study, we revealed an important role of the GP73 protein in regulating lipid homeostasis. GP73 induction in Golgi promoted SCAP/SREBP trafficking to the Golgi, leading to SREBPs activation and subsequent lipogenesis. Several lines of findings support this argument. (1) Overexpression of GP73 results in a significant activation of SREBPs and subsequent lipogenesis. (2) GP73 was shown to interact with SCAP and SREBP1. (3) GP73 overexpression led to upregulation of SCAP, enhanced SCAP-SREBPs binding and its Golgi trafficking, all are critical for SREBPs activation and lipogenesis. (4) Inhibition of GP73 in mice alleviated Tm-induced liver steatosis, in parallel with reduced SREBPs activation.

A key regulator in maintaining intracellular cholesterol levels is SCAP, which regulates cholesterol homeostasis through its interactions with SREBP-1 and SREBP-2. Regulating the stability of SCAP is another potential mechanism to modulate the lipid metabolism. The expression of SCAP was controlled by androgens and Akt kinase, while SCAP glycosylation also prolonged its half-life and enhanced its recycling between the ER and Golgi^16^. In this study, we designated GP73 as a novel regulator of SCAP. Not only does GP73 interact with SCAP in the Golgi, the expression of SCAP and its Golgi localization was also enhanced by GP73 overexpression. We also showed that the interaction between SCAP and SREBPs was greatly increased in overexpressed GP73 cells even under conditions of cholesterol sufficiency. Actually, GP73 failed to induce SREBP1 activation in the absence of SCAP. Glucose has been shown to be able to mediate N-glycosylation of SCAP and is essential for SREBP-1 activation^17^. Consistent with this, our unpublished data confirmed that glucose supplementation enhanced both SCAP and GP73 protein expressions, and subsequent SREBPs activation.

Our results from Tm-treated mice showed that administration of GP73 siRNA duplexes mitigate steatosis that was caused by typical ER stress, along with reduced SREBP1 proteolytic cleavage. Thus, GP73-induced SREBPs activation may mediate its alleviating effect upon liver steatosis. Previous studies have shown that GP73 was overexpressed in several cancers, for example, hepatocellular carcinomas, bile duct carcinomas, lung adenocarcinomas, prostate cancer and seminomas. Under a rich nutrient environment, tumor cells with GP73 overexpression can still activate SREBPs- metabolic pathway to enhance lipogenesis and promote rapid tumor growth. Specifically, in human liver cancer, our analysis of TCGA database demonstrated positive correlations between several key genes implicated in the metabolism pathway with GP73 transcripts. It remains to be elucidated whether GP73 can affect the metabolic functions of other tissues in addition to liver, for example, human obesity or type 2 diabetes.

In conclusion, our study demonstrates for the first time a pathological role of GP73 in hepatic steatosis. The increased formation of SCAP-SREBP complexes upon overexpressing GP73 provides a novel molecular linkage between GP73 and SREBP1 activation. With accumulating evidence supporting the important role of the GP73 in tumorgeneisis and cancer resistance, our study suggests an exciting potential of GP73 in cancer therapy which may help to alleviate the current drug resistance issue.

## Methods

### Western Blotting and Immunoprecipitation

Whole-cell lysates were prepared by sonication in modified RIPA buffer. 60–80 μg of protein per lane were loaded onto SDS-PAGE gels, followed by western blot analysis. When necessary, figures were cropped using Adobe Photoshop software (Adobe). Whole-cell lysates for coimmunoprecipitations were prepared by sonication in NP40 buffer (1% NP40, 150 mM NaCl and 40 mM Tris pH 7.5). Clarified lysates were then incubated with primary antibodies for 3 hrs, then with protein G-agarose (Roche) for one hour, followed by extensive wash with NP40 buffer. Supplementary Table S1 lists the primary antibodies used in this study.

### Quantitative real-time RT-PCR (QRT-PCR)

First-strand cDNA was synthesized as described previously^18^. QRT-PCR was performed in the iQ5 Real-time PCR System (Bio-Rad) using iTaq universal SYBR Green supermix (Bio-Rad). Each sample was analyzed in triplicate with GAPDH as the internal control. Supplementary Table S2 lists the primer sequences used for different genes in this study.

### Fluorescence imaging and Filipin staining

HepG2 cells were plated in 12-well plates and grown overnight on coverslips pretreated with polylysine. After washing with PBS, cells were fixed in 4% paraformaldehyde, followed by permeabilization in 0.2% Triton X-100. Permeabilized HepG2 cells were incubated with anti-GP73 at 4 °C for 12 hrs, followed by PBS wash, and 1 hour incubation with anti-rabbit, anti-goat, or anti-donkey, anti-mouse antibodies at room temperature. After further washing in PBS, the coverslips were mounted in the presence of DAPI for fluorescence visualization. For Filipin staining, cells grown on coverslips were fixed with 4% paraformaldehyde for 30 min at room temperature, followed by 2 hrs incubation in a freshly prepared Filipin III (Sigma) solution (50 μg/ml). To make fresh Filipin III solution, Filipin III was dissolved in 10 μl of dimethyl sulfoxide first, then diluted with 200 μl of PBS, and was used immediately. The UltraVIEW VoX confocal system (Perkin Elmer) was used to collect confocal images. Images were collected by sequential scanning at each excited wavelength to avoid any bleed-through between fluorophores.

### Protein binding assays

In GST pull-down experiments, cell lysates were incubated for 2 hrs at 4 °C with 5 μg purified GST or GST fusion proteins bound to glutathione beads. The glutathione beads were then washed with lysis buffer and the absorbents were subjected to SDS-PAGE and immunoblotting analysis. An aliquot of the total lysates (5%, v/v) was used as SDS-PAGE loading control.

### Plasmids and siRNA

Mammalian expression vectors encoding N-terminus Flag and Myc tagged-proteins were constructed by inserting the corresponding PCR-amplified fragments into pcDNA3 (Invitrogen). The reporter construct SREBP1-responsive element–Luc has been described previously^19^. The target sequences of siRNA for human GP73 is 5′-GCCAGUGCAUCAAUCAGAUdT-3′. The siRNA for mouse GP73 is 5′-ACCAGUGUAUCAGCCAGAUdT-3′.

### Luciferase Reporter Assays

The 1.6 kb and serial SREBP-1 promoters were PCR amplified from human genomic DNA and cloned into the pGL3 basic luciferase vector. Primers for the PCR clone is 5′-ATCTGGTACCCACCACATTCTAGGCTCAG-3′ and 5′-ATCTAAGCTTGCTCCGCGATCTGCGCCCG-3′ 293T cells were cultured in 24-well plates. Transfection was performed using Lipofectamine 2000 with 0.1 μg of reporter plasmid, 0.002 μg of the pRLTK control vector, and various amounts of DNA for each construct. The cells were harvested after 24 hrs incubation. Luciferase activity was then analyzed using Dual Luciferase Reporter Assay System (Promega). Total light production was measured with a TD-20/20 Single-Tube Luminometer (Turner BioSystems). All experiments were repeated at least three times.

### Cholesterol measurement

HepG2 cells were transfected with Flag-GP73 for the indicated times. Cellular free cholesterol concentrations were measured using an Amplex Red cholesterol assay kit following manufacturer’s instructions (Invitrogen). In brief, cells were lysed and incubated with cholesterol oxidase, horseradish peroxidase, and Amplex red in the absence or presence of cholesterol esterase. The amount of cholesterol was determined indirectly by measuring absorbance at 560 nm. The values were normalized to the total cellular protein levels, which were determined using BCA protein assay kit (Thermo Fisher Scientific).

### Animals and treatments

C57BL/6J mice at the age of 13 weeks were subjected to hydrodynamic tail vein injection of 100 μg GP73 RNAi duplexes dissolved in 1.8 ml normal saline. The injection was repeated 8 and 24 hrs later. Control mice were injected with an equal volume of normal saline or scrambled siRNA. For the Tm model, 24 hrs after the last siRNA injection, mice were injected intraperitoneally with PBS alone or Tm (2 μg/g body weight) and were killed 24 or 72 hrs later. Blood was collected and serum lipid levels were determined. Liver tissues were collected. 0.5–0.8 g liver tissues were frozen immediately in liquid nitrogen for QRT-PCR, western blot and lipid measurement. The others were fixed in neutral-buffered formalin for immunohistochemistry and frozen-fixed in OCT mounting media for Oil Red O staining. This study was approved by the Institutional Animal Care Committee of Beijing Institute of Biotechnology and all involved methods were carried out in accordance with the approved guidelines.

### Enzyme-linked Immunosorbent Assay (ELISA)

Serum of GP73 was measured using a GP73 ELISA Kit (Rejing, China) following the manufacturer’s instructions.

### Oil Red O staining

Liver tissue sections were stained with Oil Red O for lipid content according to the manufacturer’s protocol (Biovision). Sections were fixed first in formalin, followed by 60% isopropanol rinsing. Then the sections were stained with freshly prepared Oil Red O solution for 15 min, followed by 60% isopropanol rinsing and water washing before microscope analysis.

### Correlation analyses of GP73 expression with other genes

The mRNA expression and clinical data for liver, prostate and breast cancers were downloaded from the GDAC (Genome Data Analysis Center) Firehose at the BROAD Institute (https://confluence.broadinstitute.org/display/GDAC/Home). The expression values are the normalized Log2 RSEM RNA-Sequencing data^20^. Pearson correlation was calculated for expression of GP73 with expression of genes associated with ER stress signaling or metabolism pathway. The Benjamini & Hochberg^21^ adjusted p-value was calculated to measure the statistical significance of the observed correlations.

### Statistics

Statistical analysis was performed using SPSS 17.0 (SPSS Inc, Chicago, IL) and R 2.13.0 (http://www.r-project.org). All statistical tests were two-sided tests, and P values < 0.01 were considered to be statistically significant.

## Electronic supplementary material

supplementary information
