# Supplementary material for: GP73 regulates Hepatic Steatosis by enhancing SCAP-SREBPs interaction
Source: Sci Rep. 2017 Nov 2;7:14932. doi: 10.1038/s41598-017-06500-9 (PMC5668365; doi:10.1038/s41598-017-06500-9)
Supplement: Supplementary file 1 — supplementary information [file 41598_2017_6500_MOESM1_ESM.pdf]

GP73 regulates Hepatic Steatosis by enhancing SCAP-SREBPs interaction

Xiaoli Yang,[1,7] Feixiang Wu,[2,3,7] Jiankang Chen,[1,7] Cui Wang,[6,7] Qinfang Hao[1], Cuijuan Duan,[1] Li Wang,[1] Xueping Ma,[1] Deyong Zou,[1] Li Luo,[1] Yiwen Zhao,[1] Kai Guan,[2] Zirui Zheng,[2] Yuan Cao,[4] Pingping Zhang,[4] Pengyu Zhou,[1] Shengli Ma,[2] Zhifeng Yan,[5] Jia Li,[5] Yanhong Zhang,[2] Xiang He,[2] Congwen Wei,[2*] and Hui Zhong[2*]

[1] The General Hospital of Chinese People's Armed Police Forces, Beijing 100039, China.

[2] State key Laboratory of Pathogen and Biosecurity, Beijing Institute of Biotechnology, Beijing, 100850, China.

[3] Department of Hepatobiliary Surgery, Affiliated Tumor Hospital of Guangxi Medical University, Nanning 530021, People's Republic of China

[4] Department of Laboratory Medicine, The General Hospital of Jinan Military Region, Jinan, Shandong, 250031, China.

[5] The General Hospital of Chinese People's Liberation Army, Beijing, 100039, China.

[6] Shaanxi Provincial People's Hospital, Xi'an, 710068, China.

[7] These authors contributed equally to this work.

Address correspondence to: Congwen Wei(weicw@yahoo.com) or Hui Zhong (towall@yahoo.com), Beijing Institute of Biotechnology, 27 Tai-Ping Rd., Haidian, Beijing 100850, China. Phone: 861066931821.

Supplementary Information

Materials

Cell lines, cell culture and Transfection. HepG2 and 293T cell lines were purchased from the ATCC (Rockville, MD, USA). HL7702 cell line was purchased from the Chinese academy of science, Shanghai institute for cell resource center. HepG2 and 293T cells were grown in Dulbecco's modified Eagle's medium, and HL7702 cells were grown in RPMI 1640 medium. All media were supplemented with 10% fetal calf serum, together with penicillin, streptomycin, and L-glutamine. Lipofectamine 2000 reagent was used for transfection following the manufacturer's protocol (Invitrogen).

Reagents. tunicamycin (Tm), and other reagents were purchased from Sigma-Aldrich if not otherwise stated.

Supplementary Table S1: Table of antibodies

Antibody	Company	Catalog number	Dilution
GP73	Santa Cruz Biotechnology	sc-365817	1:500
SREBP1	Abcam	ab3259	1:500
SREBP2	ABclonal	A4123	1:1000
SCAP	Santa Cruz Biotechnology	sc-11355	1:500
α-Tubulin	Sigma	T6074	1:5000
GST	Proteintech	HRP-66001	1:2000
Flag	Sigma	F3165	1:5000
Myc	Sigma	M4439	1:5000

Supplementary Table S2: List of QRT-PCR primers used in this study

Name	Sequence (5'-3')
Mouse GP73	Forward: CGTCGCAGCATGAAGTCTC<br>Reverse: CAGTAGTTGAAGCCTAGCACAAT
Human GP73	Forward: TGGCCTGCATCATCGTCTTG<br>Reverse: CCCTGGAACTCGTTCTTCTTCA
Human SREBP1	Forward: ACAGTGACTTCCCTGGCCTAT<br>Reverse: GCATGGACGGGTACATCTTCAA
Human HMGR	Forward: GCTGGTAGGGAGTCAGAAGGA<br>Reverse: TGGTGGTTTTCCGGGTCTTG
Human HMGCS1	Forward: GATGTGGGAATTGTTGCCCTT<br>Reverse: ATTGTCTCTGTTCCAACTTCCAG
Human HMGCS2	Forward: GACTCCAGTGAAGCGCATTCT<br>Reverse: CTGGGAAGTAGACCTCCAGG
Human ACC1	Forward: ATGTCTGGCTTGCACCTAGTA<br>Reverse: CCCCAAAGCGAGTAACAAATTCT
Human FASN2	Forward: AAGGACCTGTCTAGGTTTGATGC<br>Reverse: TGGCTTCATAGGTGACTTCCA
Human ACSS2	Forward: AAAGGAGCAACTACCAACATCTG<br>Reverse: GCTGAACTGACACACTTGGAC

Human actin	Forward: AAGGAGCCCCACGAGAAAAT<br>Reverse: ACCGAACTTGCATTGATTCCAG
Mouse Edem	Forward: GGGGCATGTTCGTCTTCGG<br>Reverse: CGGCAGTAGATGGGGTTGAG
Mouse HMGCR	Forward: TGTTCACCGGCAACAACAAGA<br>Reverse: CCGCGTTATCGTCAGGATGA
Mouse actin	Forward: ATGACCCAAGCCGAGAAGG<br>Reverse: CGGCCAAGTCTTAGAGTTGTTG

Supplementary Figure

(S1) SREBP-1 promoter activity of HL7702 cells after transfected with Flag-vector or Flag-GP73. The luciferase activity was measured 36 hrs post transfection and normalized based on transfection efficiency.

(S2) Quantification of Filipin intensities for cells in Fig. 1j using ImageJ. All values were normalized based on those of control images.

(S3,S4) QRT-PCR analysis of HMGSC1 in HepG2 (S3) or 293T (S4) cells transfected with Flag-vector or Flag-GP73 for the 24 hrs.

(S5,S6) QRT-PCR analysis of HMGSC2 in HepG2 (S5) or 293T (S6) cells transfected with Flag-vector or Flag-GP73 for the 24 hrs.

(S7,S8) QRT-PCR analysis of ACSS2 in HepG2 (S7) or 293T (S8) cells transfected with Flag-vector or Flag-GP73 for the 24 hrs.

(S9) Immunoblotting analysis of GP73 expression in Ctrl-1, Ctrl-2, GP73-1, and GP73-2 cells transfected with Flag-GP73 or Flag-vector. α-Tubulin was used as equal

loading control.

(S10) Signal quantification of confocal immunofluorescence images shown in Fig. 4b.

(S11) Representative confocal immunofluorescence images of GP73 localized in Golgi apparatus in Ctrl-1, Ctrl-2, GP73-1, and GP73-2 cells. SCAP, red; GM130, green. DAPI : blue Scale bar: 10μm.

Cell-based studies were performed at least three independent times with comparable results. Data represent mean ± SEM. Student's t test was used for statistical analysis: **$p < 0.01$.

Supplementary Figure

S1

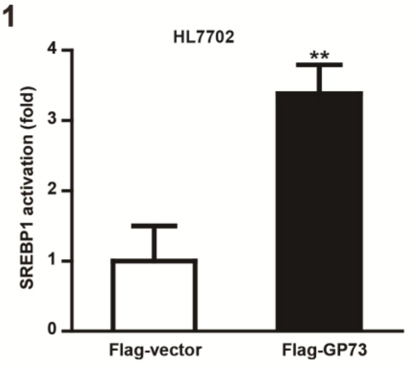

S2

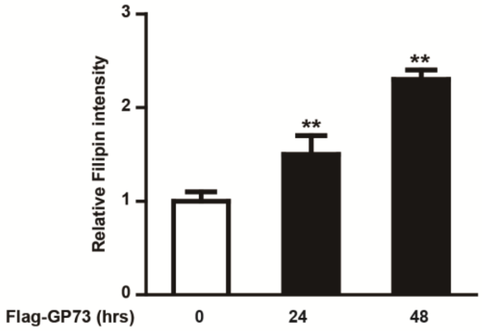

S3

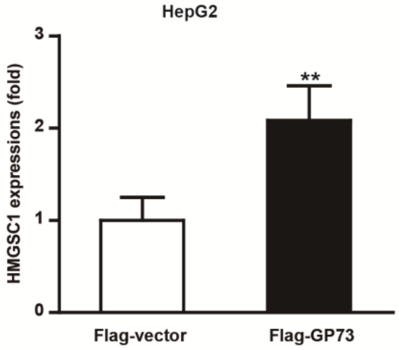

S4

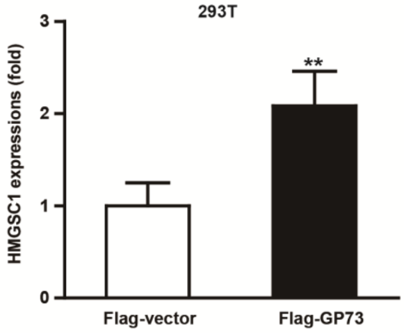

S5

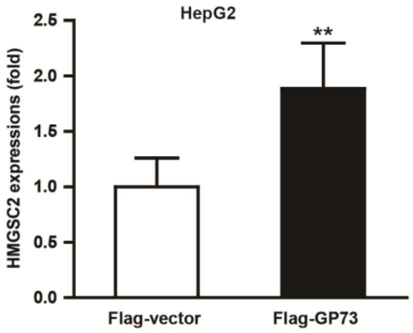

S6

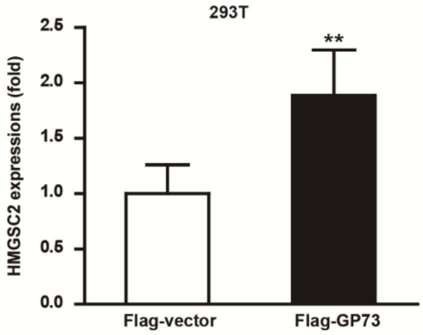

S7

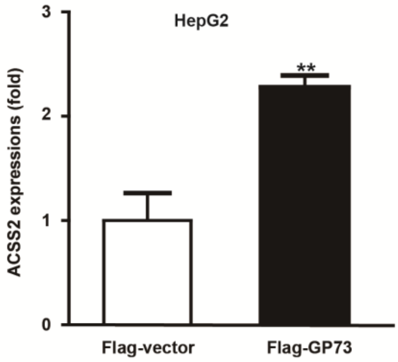

S8

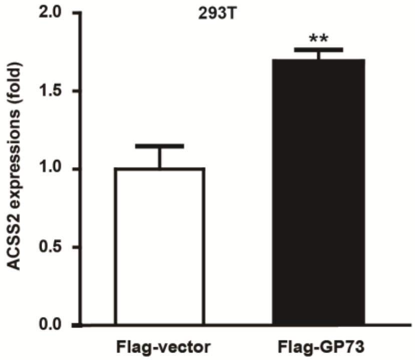

S9

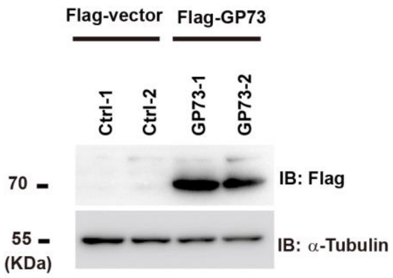

Flag-vector
Flag-GP73
Ctrl-1
Ctrl-2
GP73-1
GP73-2
70
55
(KDa)
IB: Flag
IB: α-Tubulin

S10

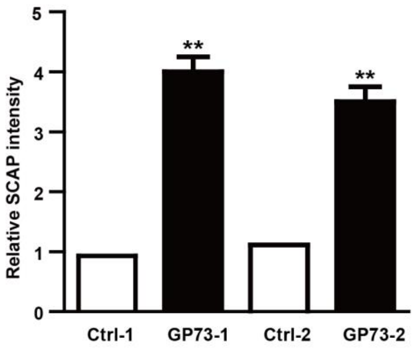

Relative SCAP intensity
0
1
2
3
4
5
**
**
Ctrl-1
GP73-1
Ctrl-2
GP73-2

S11

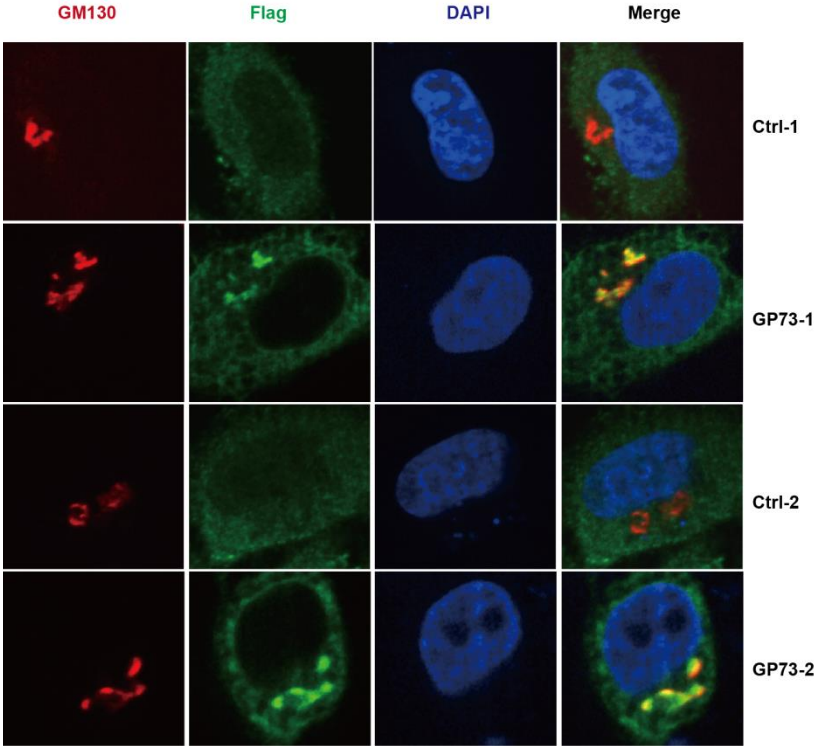

GM130
Flag
DAPI
Merge
Ctrl-1
GP73-1
Ctrl-2
GP73-2
